# Avoiding hospital-related infections during the COVID-19 epidemic

**DOI:** 10.1186/s13054-020-02929-z

**Published:** 2020-05-08

**Authors:** Jiarong Ye, Liqi Yang, Xiaotu Xi, Xinghua Lin, Deping He, Weiliang Wang

**Affiliations:** The Second Affiliated Hospital of Guangzhou University of Chinese Medicine (Guangdong Provincial Hospital of Chinese Medicine), No. 111, Dade Road, Yuexiu District, Guangzhou, 510120 China

As of April 15, 2020, the novel coronavirus disease 2019 (COVID-19) has caused over 2 million infections worldwide; in China, there have been at least 82,341 cases, over 3000 of which were in medical personnel, and at least 3342 deaths. The Guangdong Provincial Hospital of Chinese Medicine (GPHCM) is one of the largest Chinese medicine hospitals in China. In January 2020, the hospital established a new coronavirus infection pneumonia emergency prevention and control working group to train hospital staff on infection knowledge, established isolation wards to centrally treat suspected cases, and sent 88 medical personnel across nine successive medical teams to support the worst-affected area, Wuhan. Here, we summarize the steps GPHCM took to avoid hospital-related infections during the COVID-19 epidemic.

## Medical staff work protection

We established health files for each hospital employee to track their contact with the epidemic area and with suspected or diagnosed patients outside the hospital, and their relevant symptoms, such as fever and cough. We required all medical personnel to wear medical surgical masks and use appropriate hand hygiene during diagnosis and treatment activities. Wearing gloves did not and cannot replace proper hand hygiene. During daily diagnosis and treatment activities and rounds, medical staff were equipped with disposable disinfectant, a stethoscope, and disposable film gloves, and they wore work clothes, work caps, and medical surgical masks. They were advised to pay attention to the tightness of their masks and replace them after 4 h of use.

## Respiratory specimen collection and aerosol protection

Medical staff were required to wear medical surgical masks and goggles or protective masks when they collected respiratory samples from patients. Furthermore, the use of latex gloves was advised when they needed to contact patients’ blood, body fluids, secretions, or excretions. For tracheal intubation, bronchoscopy, airway care, sputum suction, and other aerosol or splash operations, medical personnel wore N95 respirators, goggles or protective screens, latex gloves, and medical protective clothing.

## Infection control in different hospital areas

We required medical personnel to standardize the personal protective equipment they wore and prohibited the wearing of personal protective equipment when leaving contaminated areas (e.g., entering lounges, dining halls) to avoid cross-infection in various areas. We kept the office area clean at all times. Areas that did not use air conditioning were kept ventilated, and those that did require air conditioning were ventilated for 30 min at least three times per day. Staff were required to wear masks when working and keep a distance of at least 1 m between people. They were also advised to wear masks and apply proper hand hygiene when attending meetings, and work clothes were not allowed to enter meeting rooms. We tried to reduce the concentrations of meetings and control the meeting times. For long meetings, we recommended opening the windows for ventilation. To avoid centralized dining as much as possible, we recommended that the medical staff use online ordering when feasible. In situations where medical staff needed to eat in the dining room, we advised hand hygiene before dining and seating separated as much as possible.

## Patient and family management

**Fig. 1:**
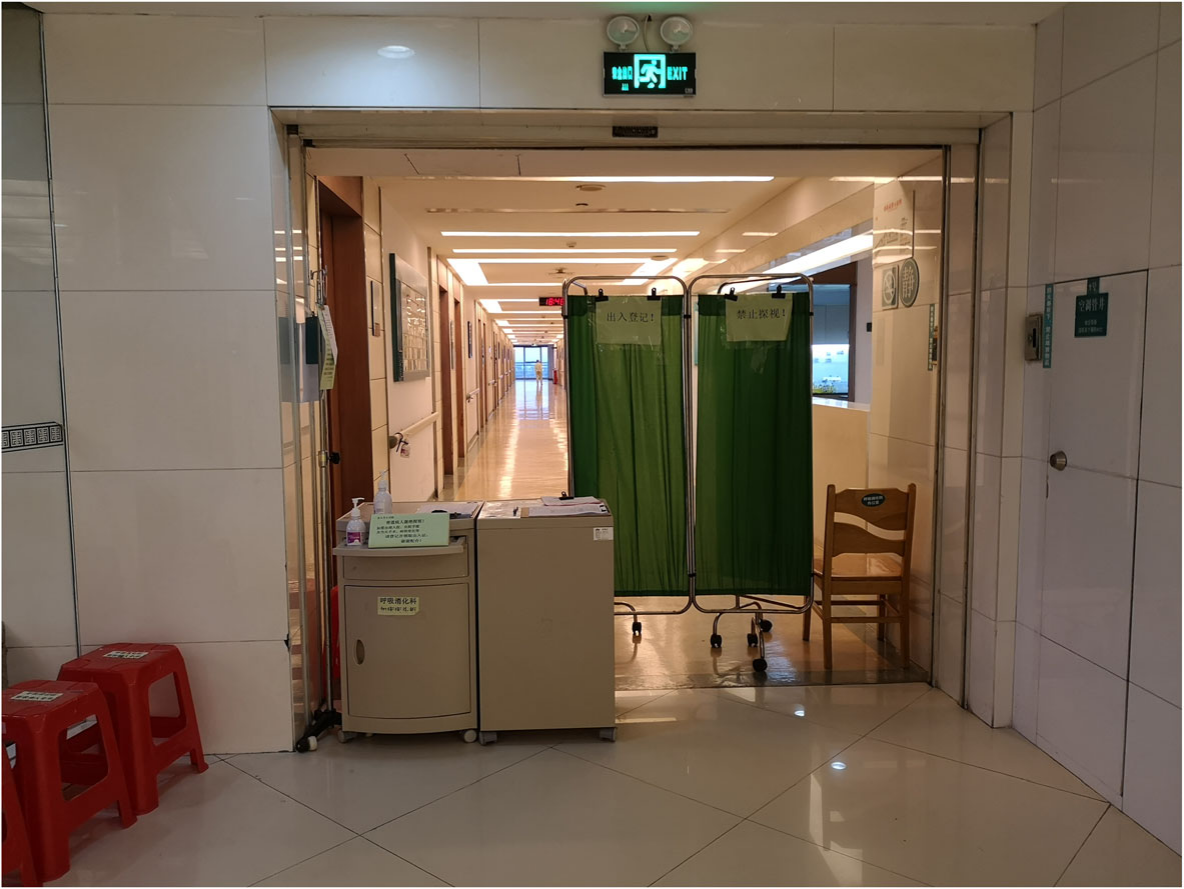
Visitation registration at the entrance and exit of the inpatient department

To ensure that COVID-19 patients did not go undetected, we performed pharyngeal swab-nucleic acid screening on all patients wanting to be hospitalized. We set up a visitation registration at the entrance and exit of the inpatient department (Fig. 1). Each patient was permitted to be accompanied by only one family member at any time. Visitors were advised to avoid staying with the patient unless it was necessary. Visiting hours were set and strictly enforced. At the time of admission, we educated patients and their accompanying family members; we also required patients and their accompanying family members to wear surgical masks.

## Emergency treatment process for suspected COVID-19 cases found in the general ward

For instances when a case of suspected COVID-19 was found in the general ward, the department director was instructed to immediately isolate the patient in question in a single room, record all close contacts, and notify all medical personnel to strengthen protective measures. The section director was required to submit the list of contact persons and the protection situation to the medical department as soon as possible.

Isolation signs stating “contact protection and droplet protection” were hung at the entrance to rooms housing suspected COVID-19 patients. These patients were required to wear medical surgical masks, and their activities were restricted to the isolation ward. Visiting by any family members who were not allowed to stay with these patients was strictly prohibited. Stethoscopes, thermometers, sphygmomanometers, and other medical appliances needed for the care of suspected COVID-19 patients were used exclusively for those individuals, and disposable medical appliances were used whenever possible. Medical equipment, appliances, and the ward environment (e.g., ground) in contact with suspected COVID-19 patients were strictly cleaned and disinfected, and the air was disinfected with ultraviolet light. The domestic waste generated by these patients was treated as infectious waste.

Suspected COVID-19 patients and medical personnel in close contact with them were tested by throat swab for SARS-CoV-2 nucleic acid as soon as possible. Medical personnel with a history of close contact were advised to go to a designated location for medical observation. The hospital logistics department registered the medical personnel under medical observation, monitored their body temperature daily, and arranged meals for them. Suspected COVID-19 cases and all close contacts who had two negative nucleic acid tests and no clinical symptoms were permitted to be released from isolation. If the suspected COVID-19 case had a positive pharyngeal swab-nucleic acid result, the ward temporarily suspended the admission of new patients. The original inpatients in the ward were isolated in a single ward and not allowed to be discharged at that time. Isolation was lifted only when two nucleic acid tests were negative. If the suspected COVID-19 patient had a positive nucleic acid test report, the hospital reported the result to the Center for Disease Control and Prevention and transferred that patient to the designated hospital for treatment.

## Data Availability

Not applicable.

